# Unmasking the Understanding of Academic Dishonesty Among Undergraduate Medical Students: "Is That Cheating?"

**DOI:** 10.7759/cureus.62609

**Published:** 2024-06-18

**Authors:** T S Gugapriya, N Vinay Kumar, Ilavenil Karunakaran

**Affiliations:** 1 Anatomy, All India Institute of Medical Sciences, Nagpur, Nagpur, IND; 2 Anatomy, Government Medical College, Palakkad, Palakkad, IND; 3 Anatomy, Karpagam Faculty of Medical Sciences and Research, Coimbatore, IND

**Keywords:** assessment, behavior, medical students, qualitative research, academic integrity policy, educational measurement, academic dishonesty

## Abstract

Introduction

Academic dishonesty threatens the environs of medical education, wherein medical graduates are expected to exhibit professional honesty. Despite the efforts of institutions and governing bodies, the implementation of an environment of academic integrity is a challenge. We hypothesized that what medical students perceive as academic dishonesty might be different from the prevalent understanding of academic dishonesty among the teaching fraternity. This exploratory study was done to identify and explore in depth what constitutes cheating in the eyes of a medical student.

Methods

This qualitative study was planned as a semi-structured interview among undergraduate medical students in the second year of study (n=25). The dimensions studied were the individual perceptions of what constitutes cheating, self-reported responses with underlying reasoning to hypothetical academic cheating scenarios, and responses on instances of self-experienced or self-observed instances of academic dishonesty.

Results

The responses indicate the ambiguous interpretation of academic honesty by students and four chief themes of the interpretation of dishonesty, based on student understanding. Our results identify core areas, such as the need for a clear and unambiguous institutional academic integrity policy, an environment of academic honesty, and strict enforcement of penalties for breach of ethical conduct, that need to be addressed to tackle the menace of academic dishonesty.

Conclusion

Themes derived from our study describe student factors, including trivialization of academic integrity, that lead to academic dishonesty. Advocacy for academic honesty in educational institutions must address these factors to enforce institutional standards.

## Introduction

Academic dishonesty encompasses a wide range of deeds of academic misconduct, including cheating in examinations, plagiarism, collaboration for individual assignments falsifying reports, and attendance irregularities [1]. Despite the explicit teaching of bioethics in medical colleges and the hidden curriculum of professionalism and ethical behavior, the prevalence of academic cheating has increased [1-3]. Authors opine that students who indulge in academic dishonesty are more prone to indulge in acts of professional dishonesty [4,5]. Moreover, studies show that students indulge in academic cheating even prior to entry into college [1,6,7].

The determinants of academic cheating in medical colleges are primarily for the purpose of "getting through" and achieving better grades [3,7,8], pressure to succeed [3,9], peer influence [10], and burnout syndrome [11]. The most cited deterrent of academic cheating has been the fear of getting caught [12]. The very act of academic cheating by its inherent immorality does not seem to be a deterrent. Academic cheating is perceived as unethical based on peer influence and the existing perception of academic cheating among peer groups [6]. Influencing factors for cheating are the prevailing hidden curriculum of professionalism within the institution, the need to be a team player and to appear knowledgeable among peers, and encouragement from residents [3,12,13].

We undertook this study to identify and explore in depth what constitutes cheating in the eyes of a second-year medical student. We intended to highlight the attitudes and unknown dimensionalities of academic cheating as perceived by undergraduate medical students in one medical teaching institution.

## Materials and methods

The study was conducted as a semi-structured interview at Chennai Medical College Hospital and Research Centre in South India, after obtaining ethical approval from the institution's Institutional Ethical Committee (approval number: CMCH&RC/IEC-149/26.11.2015). Considering the sensitive nature of the study, only students who volunteered for the study were included. All the investigators were faculty members of the Teaching Department of Anatomy, and the students had all successfully completed the first year of study (Phase I, as termed by the National Medical Commission of India). All participants were in their second year of undergraduate study (Phase II of their course as specified by the National Medical Commission of India). The convenience sample included 25 undergraduate medical students in their second year of study who were selected at random from those who volunteered, followed by a snowballing strategy. 

The guiding questions used in the interview were developed to elicit responses in three sections: (1) individual perceptions of what constitutes cheating, (2) self-reported responses with underlying reasoning to hypothetical scenarios (Table 1) that have previously been described as constituting academic cheating [1,14,15], and (3) responses on instances of self-experienced or self-observed instances of what has been considered to be academic cheating. The guide questions and vignettes used to probe for answers were decided upon by means of a consensus from faculty members in the institution, who served as educational experts.

**Table 1:** Hypothetical academic cheating scenarios that were used as guiding questions for the semi-structured interview

What do you do if…
…asked to do record work by friends/seniors?
…your classmate asks for your practical observation to copy?
…you don't get the correct set of results in a practical session?
…you were asked to say hints in an exam by your classmate?
…you were asked to show answers in exams?
…you happen to know that your classmate knows the forthcoming exam questions from a reliable source?
…your friend/classmate asks you to give "proxy" attendance for a session?
…you don't know a particular practical in an exam?

The interviews were held individually after obtaining written consent. Since participants were students, they were treated with the considerations given to a vulnerable population. Assurances of confidentiality were given, and rapport was established prior to the interview. The interviews were conducted by faculty members from the Department of Anatomy who were the participant's first-year teachers. The tone of the interview was non-judgmental, and all conversations and data were maintained confidentially. Each interview session with a participant lasted for 20-40 minutes. The data collection was either by face-to-face or by telephonic interviews. The first question pertaining to the participant's understanding of what constitutes academic cheating elicited the list of activities that students thought were included under academic cheating. Their thoughts regarding any experience of academic cheating and observation of instances of cheating (Table 2) were probed through the scenarios which were delivered verbatim in English to all participants by the investigator. The scenarios and the questions used to investigate self-reported and self-observed cheating behaviors were pre-framed (Table 2). Further probes, if needed, were conversational and included unbiased probes that would allow the investigator to gather detailed insights into the three dimensions under study. Such questions included vernacular language, wherever necessary.

**Table 2:** Guiding questions to probe for behavior in the past/in hypothetical scenarios of academic cheating

Probing questions that were used to guide the interview
Have you cheated in exams? In college/in school?
If given a situation, will you cheat?
Have you seen your classmates do the act of cheating? If so, what was your response?
Will you report such acts of cheating? If yes, do you know whom to approach?
If you were asked by your friend to be a collaborator for an act of cheating, what will you do?
Will an act of cheating hurt someone else?

The principal investigator conducted the interviews, and the transcript of the interview was documented as handwritten notes in a log book by the co-investigator. Thematic analysis was carried out, and coding of responses was done. Transcripts were read by the co-investigators individually, and themes were derived. Copies of the transcripts were made, and the chief response categories that emerged from the transcripts were underlined and placed as themes. The emergent themes were then discussed along with the principal investigator until a consensus was reached. The data are presented under the respective sections of the study and produced verbatim within quotes, where needed. Themes derived from the same are reported.

## Results

Sample demographics

The mean age of the students was 19 years, and the sample was comprised of 16 male students and nine female students (n=25) out of the total 150 students in the second year of study. Telephonic interviews were preferred by eight students, out of which six of them were female students. The other participants were interviewed face-to-face. 

Academic Dishonesty

Responses from students on what they thought constituted academic cheating fell into a single instance of cheating during examinations. These included "Exchanging answer sheets" and "In the disguise of going to the restroom, seeing answers from book/from mobile/from hidden bit."

Scenario-Based Responses

When asked to do record work by friends/seniors, the responses were mainly towards agreeing to do the work. There were negative responses also. Students who refused to do record work for others did so because they considered it as an unnecessary burden and were disinclined to do work that was not theirs. The following was the reason given by a student: "Why do I need to spend my time for them?". Students also prioritized their work above that of others, citing the lack of time. Students, who said that they would agree to do the record work, did so out of perceived peer pressure and for the sake of obliging their seniors.

For the scenario that described a classmate asking for practical observation to copy, there was a unanimous agreement in complying with the request. The thought process that students reported to this decision was that it was a friendly behavior that was expected out of them and there was nothing wrong in it, "… just to copy only."

In the scenario where students were asked what one would do if the practical results were incorrect, the majority of students said that they would manipulate the results. The reasons given for the same could be categorized as unwillingness to be questioned by faculty, an easy alternative, and inability to perceive anything wrong in the practice, "What's there in it?". A respondent however said that she would redo the practical to learn it correctly.

All respondents agreed that they would share hints about an answer during examinations with colleagues who asked. They justified their answer with reasoning that showed that they did not perceive this as cheating or did not consider it as a serious offense, "It's okay to tell hints because I am not telling the whole answer, right?". A few replied that they would help, but only when none was watching.

Responses to whether they would show their exam answer sheets for copying elicited both negative and affirmative responses. All respondents who said they wouldn't indulge in sharing answer sheets said that they were concerned about faculty reprimand. Participants who said they would help were also concerned that they would be spotted by faculty. However, there were participants who said they would "….happily show….satisfaction that I could help."

The scenario that posed a situation where "What would one do if you happened to know that your classmate knew the forthcoming exam questions from a reliable source?" also had affirmative and negative responses with variable reasoning patterns that included readiness to ask for the questions or hesitation due to a fear of refusal. Students who said they would ask for the questions gave an interesting explanation of justice saying "All or none phenomenon" and "If one knows it's unfair, but if all knows it's fair…."

The scenario regarding the proxy attendance was answered by students who thought that the issue of attendance was not to be as strictly regarded as it usually is "Just attendance ma'am. I always wonder why faculty make such a thing about attendance?"; hence, proxies could be given. The sense of mutual help was a factor in proxy attendance. Students who responded saying that they wouldn't give proxies did so either because they might "get caught" or due to "I suffered the class. I can't accept my friend enjoying attendance without pain."

Students reacted uniformly to the scenario of being allotted an examination practical exercise that they were unsure of. They responded that they would ask for help surreptitiously or directly from the faculty due to the fear of failure and that "It's ok to ask for help."

Experience and Observation of Academic Cheating

Self-reported cheating behavior at school or college was reported in 12 participants. Participants' responses varied from that they had cheated in school, but not in college, in both school and college, and neither in college nor in school. Students said that, in school, the behavior was common and, in college, they were scared of the consequences. Students, who said no for both, said they refrained from both due to fear of getting caught. Those who reported cheating in both did not think there was anything wrong with it as long as none knew. When further questioned about whether he/she would cheat in the future, if given the chance, the responses were divided with the major justifications for refusal as the fear of getting caught and the motivation for cheating as the need to pass.

Participants who answered whether they were aware of their classmates doing the act of cheating answered "yes" or "no" based on their experience. When asked further about their response or thoughts regarding the same, they answered under the following major categories of nonchalance or concern, "No, even if I see, I won't mind. It's their issue." A student was willing to discuss this with the perpetrator, "If it's by my friend, I will tell them to be careful." None of the respondents were willing to report such acts of cheating to relevant authorities. The following responses given were that it was "unnecessary trouble" and that they were friends. They also stressed that this was done in order to pass; hence, they overlooked it.

Participants also refused to be collaborators in an act of cheating even if asked to by a friend, despite their self-reported behavior of cheating. The reason cited was the fear of getting caught. Students said that they would respond with "…will tell him to study instead."

To the question of whether they thought that an act of cheating in examinations would hurt someone else, all responded that it would not hurt others; however, maybe if caught in the act, it would hurt oneself. A student responded saying "Cheating affects only those who do it."

Thematic analysis

**Figure 1:**
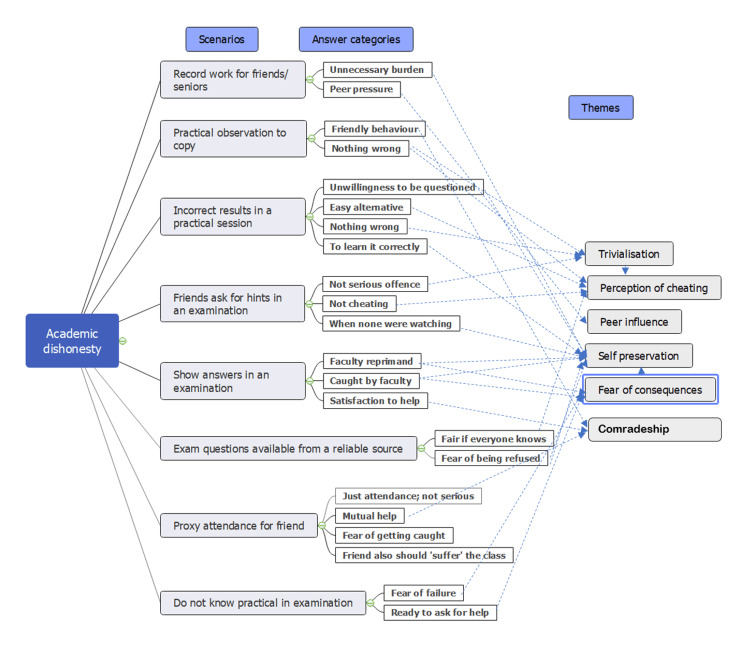
Thematic analysis coding tree showing four main emergent themes derived from the responses of the participants (n=25)

Four main themes emerged from the data transcripts, which are perception, peer influence, self-preservation, and comradeship (Figure 1). One key theme related to that of perception of academic dishonesty by the students. The responses showed that students often responded lightly and without serious concern over scenarios of academic dishonesty that were based on previous studies. Another theme that was a common thread among the transcripts was the strong sense of self-preservation. This was evidenced in responses by students who admitted to cheating and said they would cheat and also in those who refused to act dishonestly. A threshold factor for willingness to cheat as well as for avoiding academic dishonesty was based on "not getting caught," fear of faculty reprimand, and "hurt me, if caught by faculty." The motivation for carrying out acts of dishonesty also involved gain for self, such as passing an examination, getting records signed, and avoiding catching the teacher's attention.

Peer influence and the need for peer acceptance were factors at work in the reasoning behind indulging in acts of collaboration where individual work was expected and in the non-reporting of cheating incidents. Comradeship and a sense of integrity towards one's friends were themes that were encountered among the responses. The responses were worded with positive emotion of camaraderie, and any suggestion of wrongdoing was justified in light of friendly behavior.

## Discussion

We undertook the study to gather data that would better our understanding of academic cheating in the eyes of undergraduate medical students. Qualitative data was collected using a semi-structured interview among 25 volunteers using probing questions based on hypothetical scenarios and vignettes. Our results show that perception of what constitutes cheating, self-preservation, peer influence, and a feeling of comradeship were the major themes related to academic cheating. Our results identify actionable areas which can be targeted towards building academic integrity. 

Studies have reported varying definitions for cheating and behavior of cheating among medical graduates [16,17]. In this study, all participants considered only cheating during examinations within the gambit of academic cheating. This is only a single aspect of what is considered academic cheating. However, academic cheating has included cheating in examinations, aiding another student in cheating, collaborating on assignments or academic work meant to be completed independently, faking attendance, and not reporting on academic cheating [1,18]. Previous research work has regarded signing a faculty name in records, manipulation of record work, and allowing others to copy as academic cheating [8]. Students who participated in this study did not on their own state these as dishonest academic work. This reflects students' perceptions elicited as a result of the methodology used in the study. A questionnaire elicits a definite response about every aspect of academic cheating irrespective of whether the student considers the act within the repertoire of academic cheating. The methodology used in the study allows greater leeway for the student to express an opinion about what he/she considers as cheating. The interpretation of academic honesty is more consciously confined to ethical behavior at examinations. Such a discrepancy between student and faculty perceptions of what constitutes academic dishonesty and the significance attributed to such behavior has been previously reported [19]. A study on academic and professional misconduct perceptions among optometry undergraduate and postgraduate students reported that academic dishonesty was not considered a lesser evil than professional dishonesty. The study reported that more than half of the postgraduate participants did not think that allowing a classmate to copy from their paper and collaborating on individual homework was academic misconduct [20]. Our study also reveals that while students acknowledge that cheating in examinations may be considered wrong, that itself was not a deterrent to academic dishonesty during examinations. Other descriptions of academic misconduct such as manipulating practical results, faking attendance, collaborating on individual work, and such were not consciously perceived to be part of academic dishonesty even when they were specifically asked for their impression on such behavior. The perception of academic dishonesty is seen to be a major dimension for consideration because it determines the behavior that is seen as acceptable academic conduct.

The instinct of self-preservation was seen to dictate responses to experienced and hypothetical scenarios of academic dishonesty. Engaging in at least one form of cheating during their formative years along with witnessing episodes of cheating had been reported among medical students [7,21]. In our study, students reported cheating or not cheating at both school and college. Students who self-reported to have cheated in school said they did not cheat in college due to the fear of getting caught. This agrees with studies that reported that the chief deterrent for cheating in an institution was the moral policy of the institution [22] and the fear of getting caught [23]. A review on reasons for academic cheating attributes the major role played by external factors as determinants of cheating behavior [24,25]. Based on the importance of the prevailing academic environment and internal motivation in deterring academic dishonesty, it has been recommended that students must be explicitly made aware of information related to academic dishonesty [25]. The results of the present study agree with this recommendation because the perception of certain acts as dishonest and the strong instinct for self-preservation that made students wary of being "caught" by faculty were underlying themes that influenced academic cheating. Students who self-reported cheating said the main reason was in order to score better. This reason for cheating has been reported by other studies [3,7,8].

A review on academic dishonesty from India showed that medical students tend to accept many acts of cheating as "trivial" or "not cheating" [2]. Contrary to this view, studies do show that medical students considered these acts as "serious cheating" [6,10,18,26]. Reluctance to report cheating done by peers has also been observed [21]. A non-serious attitude was also reported in a study on plagiarism [26]. Participants from our study unanimously said that they would not report cheating by a peer. Students were more likely to discuss the behavior with their peers. This was also reported by another study [27]. Our study also shows that the value of peer influence and the actions associated with friendly behavior are the themes that underlie non-reporting and conformance to cheating behavior among the student body.

While the opinion expressed by students involved in this study was that cheating does not harm anyone else, it has been previously opined that academic cheating cannot be termed a "victimless crime" [28]. Learners who upheld academic ethical values, influenced by many factors, were less likely to indulge in academic cheating [29]. 

The study enables us to understand the thought processes that might explain the increase in the instances of academic cheating. Our study reveals that students do not understand academic dishonesty as broadly defined by administrative bodies. The conceptions of students regarding academic dishonesty are confined to examination malpractices. Students are responsive to the stringent measures that prevent and punish academic dishonesty. However, they are not themselves sensitized to considering academic cheating as wrong and present responses involving integrity among friends against reporting academic cheating. Peer interaction and opinion are pervasive factors in determining honest academic behavior.

This is vital to our understanding of the underlying tenacity and prevalence of academic malpractices. If universities and institutions expect to implement an atmosphere of strong academic integrity, then behavior that is considered honest and that is considered dishonest and the consequences thereof must be explicitly stated on policy documents. The same must be widely disseminated to all students and faculty through student communications, academic calendars, website displays, and notice boards. The Medical Council of India (MCI) (now superseded by the National Medical Council) implemented Competency-Based Medical Education where ethics is part of the defined curriculum [30]. The stage for open discussion and sensitization over ethical principles has been set to roll as per recent developments. The introduction of the AETCOM (Attitude, Ethics, and Communication) module could include specific modules addressing academic honesty too. The prevailing culture of an institution must be explicit in fostering strong academic values, and participative activities for students and faculty must be designed towards this end. The routine adoption of such measures along with appropriate penalties will in turn channelize peer pressure to adhere to honest practices for the well-being of the individual student. 

Limitations

While the study provided valuable insight into the attitudes and understanding of students, the results are confined to a single institution. The sampling method that was used out of the necessity of protecting students did not allow more randomization of the sample; hence, the results may be skewed with responses from like-minded participants who were willing to volunteer. The study focuses on a discrete set of acts of academic dishonesty and is therefore not inclusive of all dimensions of academic dishonesty. A study of greater sample size, wider representation of academic dishonesty, and multi-institutional representation will provide a more comprehensive outlook into the academic ethos of medical students. Our study design and the unmatched gender distribution do not allow us to comment on the influence of gender, on perceptions of academic dishonesty. Further studies on faculty perceptions may provide a comprehensive view of academic cheating. 

Recommendations

It is time for open discussions of academic dishonesty, for the purpose of clarifying acceptable behavioral standards for students, faculty, and administrators. The honor codes and academic policy in institutions need to be comprehensive and clearly stated and popularized. Activities that encourage a learning culture that is rooted in strong values of personal integrity must be designed and carried out to popularize and imbibe a deep sense of moral rectitude. Institutions might need to follow updated methods and procedures to prevent academic misconduct from being perpetrated. Penalties for academic misconduct must be transparent and stringent. Their power needs to be exploited to curb academic dishonesty, since it is seen that the prevailing perceptions and peer influence are the chief factors in the minds of students with regard to academic cheating. The focus of medical education needs to be on competency rather than grades. Capitalizing on the twin forces of peer influence and an intolerant environment for dishonesty, along with training in ethics, may be the way forward to stymie the growth of academic dishonesty in institutions and colleges. This will facilitate the training of medical professionals rooted in the values of honesty and integrity.

## Conclusions

We qualitatively explored what academic dishonesty meant to undergraduate medical students in our institution, in order to generate data to inform actions that foster academic integrity. Themes derived from student responses show a perception mismatch, with the trivialization of activities that constitute academic cheating, the influence of peer pressure, and the need for a show of comradeship. These can underlie student's indulgence in academic cheating activities. The tendency for self-preservation is one chief theme that importantly works either as a reason or as a deterrent to academic cheating. Institutional policies to maintain academic integrity must incorporate and address these factors to ensure success. 
